# A need for NAD+ in muscle development, homeostasis, and aging

**DOI:** 10.1186/s13395-018-0154-1

**Published:** 2018-03-07

**Authors:** Michelle F. Goody, Clarissa A. Henry

**Affiliations:** 1 School of Biology and Ecology, University of Maine, Orono, ME 04469 USA; 2 Graduate School of Biomedical Sciences and Engineering, University of Maine, Orono, ME 04469 USA

## Abstract

Skeletal muscle enables posture, breathing, and locomotion. Skeletal muscle also impacts systemic processes such as metabolism, thermoregulation, and immunity. Skeletal muscle is energetically expensive and is a major consumer of glucose and fatty acids. Metabolism of fatty acids and glucose requires NAD+ function as a hydrogen/electron transfer molecule. Therefore, NAD+ plays a vital role in energy production. In addition, NAD+ also functions as a cosubstrate for post-translational modifications such as deacetylation and ADP-ribosylation. Therefore, NAD+ levels influence a myriad of cellular processes including mitochondrial biogenesis, transcription, and organization of the extracellular matrix. Clearly, NAD+ is a major player in skeletal muscle development, regeneration, aging, and disease. The vast majority of studies indicate that lower NAD+ levels are deleterious for muscle health and higher NAD+ levels augment muscle health. However, the downstream mechanisms of NAD+ function throughout different cellular compartments are not well understood. The purpose of this review is to highlight recent studies investigating NAD+ function in muscle development, homeostasis, disease, and regeneration. Emerging research areas include elucidating roles for NAD+ in muscle lysosome function and calcium mobilization, mechanisms controlling fluctuations in NAD+ levels during muscle development and regeneration, and interactions between targets of NAD+ signaling (especially mitochondria and the extracellular matrix). This knowledge should facilitate identification of more precise pharmacological and activity-based interventions to raise NAD+ levels in skeletal muscle, thereby promoting human health and function in normal and disease states.

## Background

The laws of thermodynamics state that energy remains constant within a closed system. Energy is neither created nor destroyed; it just changes form. In biology, the flow of energy through organisms, tissues, and cells is a universal requirement to sustain life. Energy in the form of dietary nutrients is metabolized into adenosine triphosphate (ATP) and other molecules that power necessary cellular processes. Therefore, energy is potentially the most important information that cells interpret. As such, it would logically follow that multiple, highly nuanced cellular mechanisms for nutrient and energy sensing would evolve due to intense selective pressures. This exquisite tailoring of form to function is perhaps best exemplified in the mitochondrial reticulum that facilitates quick energy transfer throughout the muscle cell [1]. Oxidized nicotinamide adenine dinucleotide (NAD+) and its reduced form, reduced nicotinamide adenine dinucleotide (NADH), are critical molecules involved in energy generation because NAD+/NADH participate in oxidation-reduction (redox) reactions in the tricarboxylic acid (TCA) cycle. In this context, NAD+ is not destroyed, it is reversibly reduced. However, NAD+ is also a cosubstrate in multiple post-translational modifications such as deacetylation and ADP-ribosylation. These reactions result in consumption of NAD+, necessitating replenishment of cellular NAD+ stores. Thus, maintaining the balance between NAD+ degradation and biosynthesis is extremely important for cellular homeostasis. In this review, we will discuss evidence and mechanisms of NAD+ function in skeletal muscle development, homeostasis, and disease.

In addition to mediating posture and locomotion, skeletal muscle is a metabolically important organ. Skeletal muscles sense, produce, store, and utilize nutrients and energy transfer molecules that enable muscle to participate in systemic, energy-expensive processes such as temperature regulation and immunity. Skeletal muscle is plastic and readily adapts to change. Examples of skeletal muscle plasticity include hypertrophy in response to weight bearing exercise, atrophy in response to disuse, and fiber type switching due to changes in gene expression, cellular metabolism, or innervation. It seems likely that there is coordination between skeletal muscle’s energy use/sensing and the signaling pathways that regulate its remarkable phenotypic plasticity.

Muscle diseases have a negative effect on health, lifespan, and/or quality of life. Genetic muscle diseases caused by mutations result in a variety of ultrastructural defects in muscle cells and progressive loss of muscle mass and function via multiple different mechanisms. Inflammatory or metabolic diseases (such as diabetes, obesity, autoimmune diseases, cancer, and infections) can result in loss of skeletal muscle as well. Given the integration and interdependence of the nervous and muscular systems, neural disorders or injuries can also impair muscle tissue structure and function. Additionally, skeletal muscle is lost as a natural part of the aging process, and this loss is exacerbated in a condition called sarcopenia. Adequate skeletal muscle mass prior to illness, injury, or aging is predictive of recovery and healthy aging (reviewed in [2]). Skeletal muscle performs a diverse set of crucial functions in organisms and promoting muscle health and exploiting its plasticity could improve locomotion, metabolism, and quality of life in many disorders.

In this review, we will discuss the recent data that document conserved roles for NAD+ in skeletal muscle development, regeneration, aging, and disease as well as interventions targeting skeletal muscle and affecting NAD+ that suggest promising therapeutic benefits. We will also highlight gaps in our knowledge and propose avenues of future investigation to better understand why and how NAD+ regulates skeletal muscle biology.

## Introduction to NAD+

**Fig. 1:**
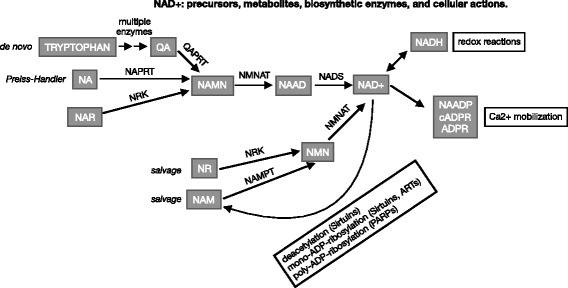
NAD+ biosynthetic pathways. NAD+ can be synthesized via de novo, Preiss-Handler, and multiple salvage pathways. NAD+ precursors and metabolites are represented in gray boxes. The enzymes involved in each conversion are listed above the arrows. The cellular outputs regulated by NAD+ or metabolites are listed in white boxes with black outlines. For explanation of the acronyms, please see the abbreviations list in the main body of the text

A role for NAD+ in cellular ATP production has been known for approximately a century. NAD+ was originally referred to as “cozymase” when first discovered as a hydrogen and electron acceptor and donor in redox reactions (reviewed in [3]). The metabolism of glucose in cells that can occur in either the presence or absence of oxygen (aerobic/oxidative vs. anaerobic/glycolytic, respectively) requires NAD+. NAD+ plays roles in multiple, diverse cellular processes in addition to redox reactions. NAD+ is a cosubstrate in deacetylation reactions as well as in mono- and poly-ADP-ribosylation. While NAD+ can cycle between its oxidized and reduced forms in redox reactions, it is cleaved when it functions as a cosubstrate. Thus, NAD+ must be constantly re-synthesized from dietary precursors in order to maintain cellular pools of NAD+ as well as appropriate NAD+/NADH ratios (Fig. 1). It is not entirely clear whether the critical aspect of NAD+ biology in skeletal muscle is levels of NAD+ or the NAD+/NADH ratio [4]. For simplicity, we will generally just refer to NAD+ levels.

Given the large demand for NAD+ as a cosubstrate in many cellular reactions, it has been estimated that grams of NAD+ precursors would need to be ingested daily in order to maintain physiological NAD+ concentrations [5]. As the recommended daily allowance of dietary NAD+ precursors is only in the milligram range, this suggests that there are multiple biosynthetic pathways that generate NAD+ from different dietary precursors and that the nicotinamide byproduct formed upon NAD+ cleavage is efficiently recycled for NAD+ biosynthesis [6].

NAD+ can be formed from dietary ingestion of the amino acid tryptophan or forms of vitamin B3/niacin (nicotinic acid, nicotinamide, and nicotinamide riboside). Quinolinic acid, a metabolite of tryptophan, can also be a starting point for NAD+ biosynthesis (Fig. 1). The de novo pathway of NAD+ biosynthesis from tryptophan involves seven enzymatic steps. In contrast, the salvage pathways of NAD+ biosynthesis from niacin involve only two or three enzymatic steps. Important enzymes involved in NAD+ biosynthesis are nicotinamide phosphoribosyltransferase (NAMPT), which acts on nicotinamide; NRK1 and/or NRK2, which act on nicotinamide riboside; and the NMNAT family of enzymes, which act on multiple substrates in de novo and salvage NAD+ biosynthesis (Fig. 1).

### Conserved roles for NAD+ as a mediator of health

NAD+ is linked to healthspan and lifespan in organisms separated by millions of years of evolution. There is a conserved decline in the NAD+/NADH ratio as worms, mice, and humans age [7–9]. Lower levels of NAD+ and lower NAD+/NADH ratios are correlated with functional decline and diseases of aging and mitochondrial dysfunction, such as metabolic and neurodegenerative diseases. Enhancing NAD+ levels is sufficient to increase lifespan in worms [7, 10], and in worm and mouse models of ataxia telangiectasia [11]. NAD+ levels are further implicated in human health in that dietary deficiencies in NAD+ precursors can cause pellagra. Pellagra results in skin, gastrointestinal, psychological, and neurodegenerative symptoms and is fatal without B vitamin or other appropriate NAD+ precursor supplementation. Given the conserved decline of NAD+ with age and the ability of NAD+ to increase lifespan in worms, it has been hypothesized that the loss of skeletal muscle mass and function due to aging or disease could be caused, at least in part, by lower availability of NAD+. We will review roles for NAD+ in muscle development, homeostasis, disease, and aging and highlight interventions that restore NAD+ levels and ameliorate muscle dysfunction.

### NAD+ compartmentalization in skeletal muscle

Skeletal muscle is energetically expensive. Skeletal muscle cells take in glucose and store it as glycogen. When there is a need for more energy within muscle fibers, glycogen is converted to glucose that is used to generate ATP. Skeletal muscle cells can contain thousands of mitochondria, which are where the aerobic/oxidative metabolism of glucose occurs. As NAD+ is required as a hydrogen and electron acceptor and donor for aerobic cellular metabolism, NAD+ localization to mitochondria is important for muscle function. Mitochondria are the subcellular compartments that have the highest level of NAD+ in skeletal muscle cells. However, 95% of the NADH in skeletal muscle is also localized to mitochondria. Therefore, the NAD+/NADH ratio is relatively low in skeletal muscle mitochondria despite there being high levels of NAD+ [12–14]. The observation that mitochondria contain the greatest amount of NAD+ in skeletal muscle cells reflects the prioritization of redox and ATP-generating reactions for muscle biology; however, NAD+ is involved in many cellular reactions as discussed below.

**Fig. 2:**
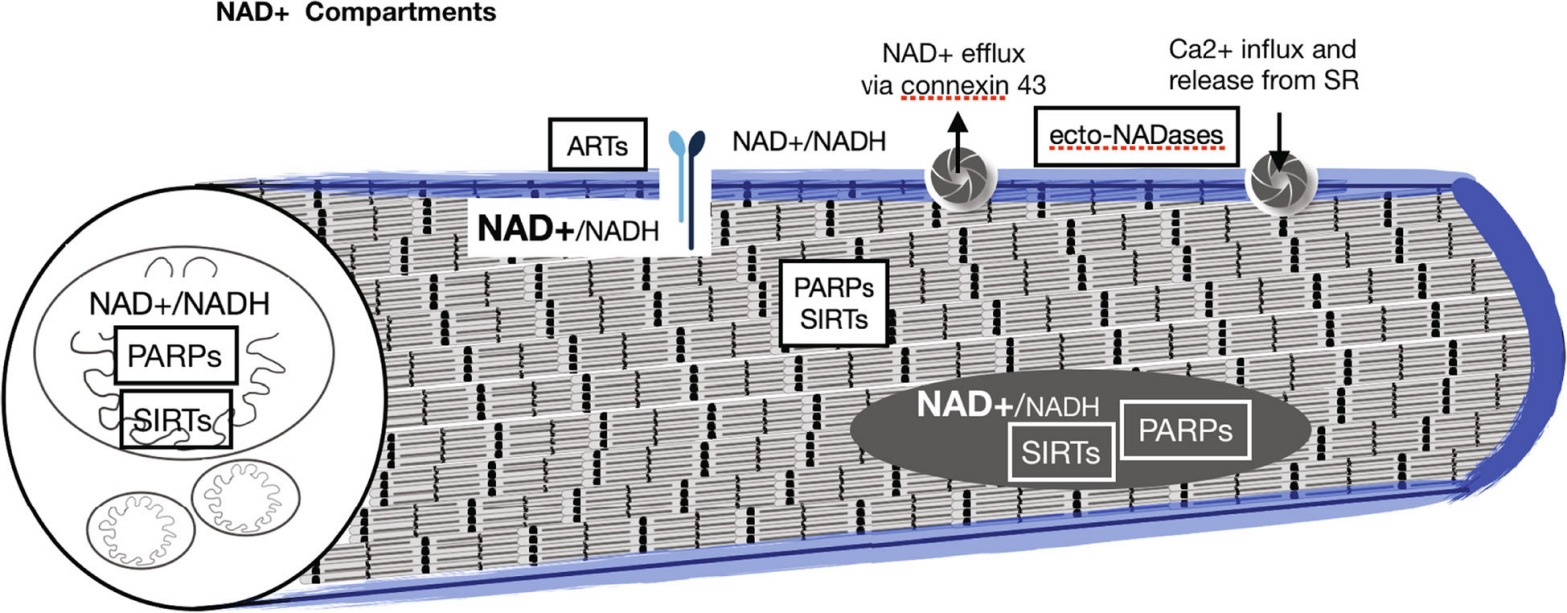
Compartmentalization of NAD+ pools in skeletal muscle. Diagram of a striated skeletal muscle fiber. NAD+ is localized to mitochondrial, nuclear, cytosolic, and membrane proximal pools in muscle cells. Additional NAD+ compartments not diagrammed here include vesicular compartments. The NAD+/NADH ratio is higher in the nuclear and cytosolic compartments compared to the mitochondrial compartment. The ratio is unknown in the membrane proximal compartment in muscle. Enzymes that consume NAD+ and their relative subcellular localizations are found within black or white boxes. Integrin receptors and membrane channels that transport NAD+ and calcium across the sarcolemma can be seen in the diagram

NAD+ localization to other organelles facilitates the additional NAD+-consuming reactions that occur in muscle homeostasis (Fig. 2). A cytosolic pool of NAD+ participates in the anaerobic/glycolytic metabolism of glucose into ATP. This type of cellular metabolism is more rapid but less efficient in ATP generation than the aerobic/oxidative metabolism that occurs in mitochondria. Metabolites of cytosolic NAD+, such as nicotinic acid adenine dinucleotide phosphate (NAADP), cyclic ADP-ribose (cADPR), O-acetyl-ADPR (OAADPR), and ADPR, regulate calcium ion channels which can promote calcium influx from the extracellular environment or calcium release from internal stores (reviewed in [6, 15, 16]), (Fig. 2). Like ATP, calcium ions are required for functionality of the contractile apparatus in striated muscle and calcium ions are also important secondary messengers in signal transduction events. Considering that skeletal muscle evolved a subcellular organelle specifically designated for storage and rapid release of calcium ions (the sarcoplasmic reticulum), the role that NAADP, cADPR, OAADPR, and ADPR play in regulating calcium mobilization in skeletal muscle is a burgeoning area of research.

In the nucleus, NAD+ is used as a cosubstrate for sirtuin-mediated deacetylation and mono-ADP-ribosylation reactions as well as poly-ADP-ribosylation reactions carried out by PARP enzymes (Fig. 2). Sirtuins regulate gene expression and protein function via deacetylation of histones or other proteins, respectively. Sirtuin activity is generally beneficial for cellular and organismal health [7, 17]. PARP activity is stimulated by oxidative stress [18, 19]. PARPs are involved in repair of DNA damage. PARP-mediated repair of DNA damage is certainly a beneficial process in times of nutrient excess and oxidative stress; however, PARP activity drastically reduces the pool of nuclear NAD+ and essentially outcompetes sirtuins for their shared cosubstrate. Therefore, the reduction of oxidative stress and the genetic or pharmacological inhibition of PARPs can increase the NAD+ available for sirtuin-mediated deacetylation reactions. This has shown to be a promising therapeutic approach for some muscle conditions [20–23].

**Fig. 3:**
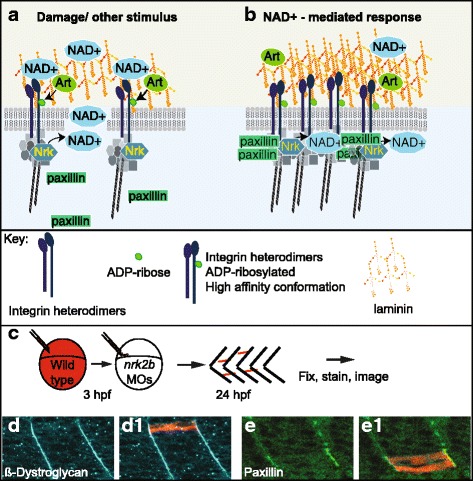
Hypothesized mechanism of membrane proximal NAD+ action in muscle-ECM adhesion. (**A**–**B**) Model of membrane proximal NAD+ regulation of subcellular protein localization, post-translational modification, laminin-binding affinity, and ECM organization in muscle. (**A**) Damage scenario. NRK enzymes localize to cytoplasmic tails of Integrin receptors for laminin and generate an intracellular membrane proximal pool of NAD+. In response to damage, intracellular membrane proximal NAD+ generated by NRK enzymes is actively transported or leaks across the sarcolemma into the extracellular environment. Extracellular membrane proximal NAD+ is consumed by ART enzymes as ADP-ribose moieties are added to Integrin receptors for laminin in mono-ADP-ribosylation reactions. Intracellular localization of Paxillin to cell-matrix adhesion complexes is disrupted. Extracellular organization of laminin is disrupted. (**B**) NAD+-mediated damage response scenario. Due to the movement of intracellular membrane proximal NAD+ to the extracellular environment, ADP-ribosylation of Integrin receptors for laminin change to a high-affinity binding conformation. Laminin organization is increased, Paxillin localization to cell-matrix adhesion complexes is restored, and Integrin-laminin binding is augmented. (**C**–**E**) Reprinted with permission from [33]. **c** Model of genetic mosaic experiment to determine if wild-type Nrk2b is sufficient in a cell autonomous manner for subcellular localization of beta-Dystroglycan and/or Paxillin to cell-matrix adhesions. Fluorescent dextran labeled wild-type cells were transplanted into a Nrk2b-deficient background and subcellular localization of beta-Dystroglycan and Paxillin were determined by immunohistochemistry. (**D**–**E**) Side mount, anterior left, 26 hpf *nrk2b* morphant hosts with transplanted wild-type cells (red) and beta-Dystroglycan (blue) or Paxillin antibody staining (green). (**D**-**D1**) Beta-Dystroglycan robustly localizes to MTJs in *nrk2b* morphants in the presence and the absence of transplanted wild-type cells. (**E**-**E1**) Robust Paxillin localization to cell-matrix adhesion complexes in *nrk2b* morphants is rescued in the transplanted wild-type cells, but not in the Nrk2b-deficient cells surrounding them. Therefore, Nrk2b is cell autonomously sufficient for subcellular localization of Paxillin, likely through generation of membrane proximal NAD+

Adhesion of muscle cells to their extracellular matrix (ECM) microenvironment is critical for muscle development and homeostasis (reviewed in [24]). Muscle fibers, muscle fiber bundles, and whole muscle are surrounded by ECM. One substructure within the ECM is the basement membrane. Basement membranes are specialized parts of the ECM that serve a myriad of functions, playing both structural and signaling/organizational roles. Laminin, a major component of basement membranes, is necessary for basement membrane assembly [25]. NAD+ is found in the extracellular environment surrounding cells [6, 16], where it is a cosubstrate for ecto-ADP-ribosyltransferases. NAD+ is important for normal structure of the extracellular microenvironment (Fig. 3). There is a membrane tethered, extracellular mono-ADP-ribosyltransferase, ART1, that ADP-ribosylates Integrin alpha7 (ITGA7) at multiple sites in a NAD+ concentration-dependent manner [26–30]. This ADP-ribosylation by ART1 is not readily reversible and leads to an increased laminin-binding affinity of Integrin alpha7beta1 in differentiated myotubes. Thus, this ADP ribosylation event might be a protective mechanism that increases adhesion to laminin when the sarcolemma has been compromised and NAD+ leaks into the extracellular space [27]. Another example of NAD+ function in the plasma membrane proximal compartment of muscle cells involves NRK enzymes. Yeast and human NRK enzymes participate in an alternative salvage pathway that generates NAD+ [31]. NRK2 (aka MIBP2, ITGB1BP3) was found to bind to the cytosolic tail of Integrin beta1 and regulate adhesion of cultured mouse muscle cells to laminin-111 but not fibronectin [32]. We found that zebrafish Nrk2b localizes to the myotendinous junction and is required for proper organization of laminin-111 in the developing myotendinous junction. Exogenous NAD+ rescues laminin integrity at the myotendinous junction in Nrk2b-deficient zebrafish [33], indicating that zebrafish Nrk2b also functions to generate NAD+. Laminin organization at the myotendinous junction is a novel role for NAD+ and the mechanism is still unknown. Taken together, the above data suggest the potential of an additional pool of NAD+, the membrane proximal pool, that mediates skeletal muscle physiology (Fig. 3).

NAD+ is also localized to vesicular compartments within cells, such as the Golgi, endoplasmic reticulum, peroxisomes, and lysosomes. It is tempting to speculate that compartmentalization of NAD+ allows for finer spatial and temporal control of NAD+-dependent signaling. To our knowledge, roles for NAD+ in function of these organelles in skeletal muscle have not been elucidated and this represents a major area for future research. We will focus on new data implicating the membrane proximal, mitochondrial, and nuclear/cytoplasmic pools of NAD+ in skeletal muscle development and health.

## NAD+ in muscle development and regeneration

**Fig. 4:**
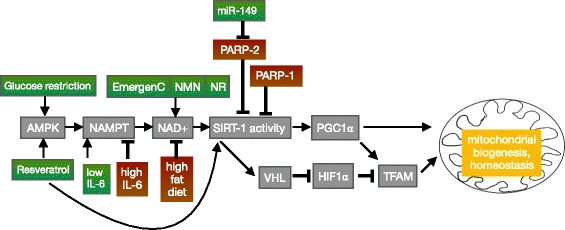
Signaling pathways by which NAD+ regulates mitochondrial biogenesis and homeostasis in muscle. Arrows denote promotion and “T”s signify inhibition of mitochondrial biogenesis or homeostasis. Interventions in green boxes promote NAD+ levels and interventions in red boxes deplete NAD+ levels, thereby regulating mitochondria number and function. For explanation of the acronyms, please see the abbreviations list in the main body of the text

Periods of muscle development and/or growth may be coordinated with nutrient availability. As NAD+ provides cells with information about nutrient levels, it is likely that NAD+ is involved in regulating these cellular transitions. A decrease in NAD+ levels is observed in myotubes compared to myoblasts (both in C2C12s and primary muscle cells) [34]. Thus, the current thinking is that decreased NAD+ levels are permissive for muscle differentiation. The regulation of muscle differentiation by NAD+ in cultured mouse myoblasts seems to be mediated by SIRT1 activity. SIRT1 represses muscle differentiation: SIRT1 complexes with myoD and the acetyltransferase p300/CBP-associated factor (PCAF) to inhibit muscle gene transcription in vitro [34]. Fulco and colleagues hypothesized that nutrient sensing of glucose would be integrated with NAD+ levels because this would provide a mechanism where nutrition and myogenesis could be coordinated [4]. They found that energy sensing feeds into NAD+ levels by modulating the levels of NAMPT. NAMPT activity is promoted by AMPK, which is increased with glucose restriction. Thus, glucose restriction activates NAMPT activity, resulting in increased NAD+, increased SIRT1 activity, and repression of muscle differentiation [4] (Fig. 4). There is no evidence that primary (embryonic) myogenesis requires modulation of NAD+ levels in vivo. There is, however, data indicating that secondary (fetal) myogenesis may integrate nutrient sensing with NAD+ levels and SIRT1 activity to mediate muscle growth in vivo. Rats born to calorie-restricted mothers are smaller due to deficits in secondary myogenesis [35]. These data support that muscle differentiation and growth are linked to nutrient availability via NAD+. However, the mechanisms regulating which signal transduction cascades and which protein-protein interactions are invoked downstream of NAD+ inputs are unclear. There are additional unanswered questions regarding roles for NAD+ in modulating SIRT1 activity during muscle development and growth, such as how are NAD+ levels decreased to alleviate SIRT1-mediated repression of differentiation, and how are NAD+ levels restored after differentiation?

One factor that may contribute to changes in NAD+ levels in the transition from myoblasts to myotubes is PARP-1 (Fig. 4). PARP-1 covalently attaches branched ADP-ribose chains to proteins using NAD+ as a substrate. Thus, PARP-1 is a major NAD+ consumer and NAD+ levels tend to be inversely related to PARP-1 levels in multiple cell types. This phenomenon has been observed in C2C12 myotubes exposed to oxidative stress. H2O2 activated PARP-1 and a subsequent decrease in NAD+ levels were observed [23]. Decreased NAD+ did not reduce SIRT1 protein expression; however, SIRT1 activity, as measured by PGC1-alpha acetylation, was reduced [23]. PARP-1 levels are high in myoblasts and lower in myotubes (C2C12 and a rat skeletal muscle line L6) [22]. Thus, changing levels of PARP-1 likely play a role in SIRT1 activity during muscle differentiation via modulation of NAD+ levels.

An additional effect of PARP-1 levels is sensitivity to oxidative stress. PARP-1 is induced by oxidative stress and plays a role in DNA repair. However, if left unchecked, PARP-1 activity depletes NAD+ levels. This adversely affects electron transport and ATP synthesis and can lead to necrosis. It is also possible that the downregulation of PARP-1 levels in developed myotubes provides a mechanism for increased resistance to oxidative stress [22]. Inhibition of PARP-1 catalytic activity increased protection against oxidative stress in C2C12 myotubes and overexpression of PARP-1 sensitized these cells to oxidant-induced injury. Thus, in the future it will be interesting to determine mechanisms regulating PARP-1 expression during muscle differentiation, to ask whether PARP-1 plays a significant role in differentiation by modulating NAD+ levels, and, if so, whether PARP-1 interacts with nutrient sensing by AMPK.

One question is whether muscle development and muscle repair show similar responses to NAD+ levels during differentiation. Muscle repair is mediated by muscle satellite cells that reside next to muscle fibers under the basement membrane [36, 37]. These self-renewing cells are usually quiescent, but they proliferate and differentiate in response to muscle injury. Recent data suggest SIRT1 plays a role in actively maintaining quiescence in satellite cells by repressing transcription of myogenic genes. Quiescent satellite cells use oxidative-phosphorylation (OXPHOS) to generate energy [38]. Metabolic reprogramming is a hallmark of satellite cells, and Ryall and colleagues asked whether these cells shifted their metabolic state when activated. They observed that satellite cells switch from primarily OXPHOS to increased glycolysis and glutaminolysis when activated and proliferating [38]. One consequence of this metabolic shift is that, similar to muscle development, NAD+ levels decrease, SIRT1 activity decreases, and this change is permissive for myogenic gene transcription [38]. Inactivation of SIRT1 in satellite cells results in initially smaller mice with smaller muscle fibers at postnatal day 9, although the mice recover by postnatal day 14 [38]. In the future, it would be quite interesting to determine the mechanism of size recovery. Taken together, these data indicate that muscle repair and muscle development both rely on changing NAD+ levels and that this process mechanistically links metabolism/energy availability with differentiation in both contexts.

### Membrane proximal NAD+ in muscle development

The ECM protein laminin is necessary for primary myotome development. In mice, laminin is thought to act as a barrier that keeps myogenic cells inside the myotome. When laminin polymerization is compromised, myocytes disperse into the sclerotome region [39]. Similarly, in *lamb1* or *lamc1* mutant zebrafish, some fast-twitch muscle cells extend into adjacent myotomes rather than stopping at myotendinous junctions [40]. Surprisingly, NAD+ biosynthesis plays a role in potentiating laminin organization in the basement membrane at the myotendinous junction. Zebrafish deficient for Nrk2b exhibit defects in laminin organization that are rescued with exogenous NAD+ supplementation. Although the mechanism is not known, NAD+ appears to act via the laminin receptor Integrin alpha6 and promotes subcellular localization of Paxillin to cell adhesion complexes [33] (Fig. 3). This suggests that, at least in zebrafish muscle cells, the NAD+ synthesized in the membrane proximal compartment during muscle development modulates the ECM. Interestingly, there were no observed muscle defects in NRK2 knockout mice [41]. Analysis of NRK1 and NRK2 single and double knock out cells in a primary muscle culture system demonstrated some redundancy between NRK1 and NRK2 in salvage NAD+ biosynthesis from NR [41]. It is possible that the functional redundancy between NRK1 and NRK2 observed in mouse muscle cells does not occur in zebrafish and could explain the discrepancy in muscle development phenotypes between the two species. Regardless of this difference, NAD+ was subsequently implicated in *C. elegans* muscle development and function [42] and in mouse skeletal muscle health [43–46]. Therefore, a role for NAD+ biosynthesis in muscle development and function appears to be well conserved.

## NAD+ and aging muscle

**Fig. 5:**
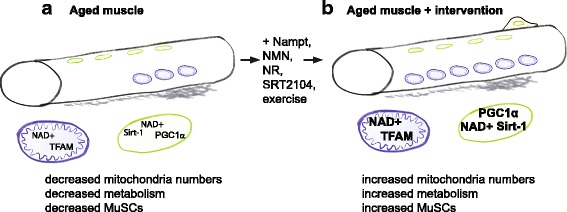
Model of the pathological observations in aged muscle and their reversal by augmentation of nuclear and mitochondrial NAD+ levels. **a** Fewer mitochondria, fewer muscle stem cells, reduced levels of NAD+ in nuclei and mitochondria, and reduced nuclear SIRT-1 activity have been observed in aged muscle. **b** Repletion of NAD+ levels in nuclei and mitochondria via various interventions increases mitochondria, increases muscle stem cells, and increases nuclear and mitochondrial NAD+ levels thereby increasing SIRT-1 activity. One mechanism involves nuclear SIRT-1-mediated regulation of TFAM and PGC1alpha gene expression and their effect on mitochondrial metabolism

Aging is considered by some to be the tail end of development. If changes that occur throughout the lifespan are thought of as part of a developmental continuum, then it logically follows that developmental robustness and plasticity likely impact the aging process. The loss of skeletal muscle mass and function with aging is called sarcopenia. Sarcopenia decreases quality of life and increases the risk of metabolic diseases. Although there is some controversy regarding whether age decreases mitochondrial content and ATP production (reviewed in [47]), the data in general seem to suggest that mitochondrial dysfunction is a major driver of muscle aging [48–52] (Fig. 5). As described above, mitochondrial homeostasis is strongly influenced by NAD+ via SIRT1. One key target of SIRT1 is PGC-1alpha, which promotes mitochondrial biogenesis [53]. SIRT1 also promotes mitochondrial biogenesis by potentiating mitochondrial transcription of oxidative phosphorylation genes in a PGC-1alpha-independent manner [54]. This pathway involves stabilization of HIF-1alpha, and AMPK appears to mediate the switch between these two SIRT1-dependent pathways of mitochondrial biogenesis [54]. NAD+ levels are reduced in aged muscle in rats, mice, and humans [9, 54, 55], leading to reduced SIRT1 activity, and reduced mitochondrial homeostasis. The mechanisms by which NAD+ levels are modulated are of extreme interest because understanding these mechanisms could lead to approaches to slow or even halt the loss of muscle mass and function during aging. Here, we will discuss recent insights into the mechanisms underlying decreased NAD+ with aging and highlight interventions that increase NAD+ and skeletal muscle function.

### NAMPT levels decrease during aging, increasing NAMPT improves aged muscle

In aged rats, NAD+ levels have been shown to decrease due to lower levels of the rate limiting enzyme in NAD+ biosynthesis, NAMPT [56]. Knock out of NAMPT in skeletal muscle of mice (mNKO mice) demonstrated that NAMPT is required for muscle function [45]. NAD+ and ATP levels were decreased by 85 and 60%, respectively, in mNKO mice. At 3 months, functional defects were not immediately apparent on a treadmill test: mNKO mice tired at similar rates to controls. However, when extensor digitorum longus (EDL) muscle was repeatedly electrically stimulated ex vivo, force generation was significantly reduced [45]. This deficiency, apparent in response to repeated electrical stimulation, could be because mitochondria have been found to recover more slowly in aged vs young mice, at least in whole lumbrical muscle [49]. mNKO mice were only slightly smaller than wild-type siblings; however, histological changes (smaller fiber diameter, increased Evans blue infiltration, and increased central nuclei) were apparent at 3 months. These phenotypes worsened through time and were classified as “NAD+-deficient myopathy” [45]. Thus, mNKO mice appear to be a good model for investigating the effects of reduced NAD+ on skeletal muscle biology in the context of aging on a developmental continuum.

NAMPT is not only necessary for muscle function, it is sufficient to combat the decline in muscle performance with aging. Overexpression of NAMPT in cardiac and skeletal muscle throughout life increased healthspan [45]. Overexpression of NAMPT in skeletal muscle resulted in improved exercise capacity in aged mice. NAMPT overexpression increased NAD+ levels and, because of this higher baseline, NAD+ levels in aged mice overexpressing NAMPT in skeletal muscle were similar to those of young control mice [45]. This was despite NAMPT overexpressing mice still experiencing a decline in NAD+ levels with aging. Therefore, interventions that bolster NAD+ levels prior to and during aging, such as NAMPT overexpression, maintain the functional capacity and promote the healthy aging of skeletal muscle.

So how can NAMPT levels be increased during aging? Multiple studies in three different mammals indicate that exercise increases NAMPT. Exercise training increased NAMPT levels, NAD+, and SIRT1 activity in aged rats [56]. NAMPT protein was increased 16% in humans in the leg that conducted one-legged knee-extensor exercises compared to the untrained leg [57]. This result is quite compelling, but it suggests that resistance training with the goal of increasing NAMPT expression would need to target all muscle groups. A similar increase in NAMPT expression was observed in the quadriceps of endurance-trained mice (this was the only muscle examined) [57]. Electrostimulation of primary skeletal muscle cells in vitro also increased NAMPT expression [58]. One study found that exercise training increased SIRT3 expression but not NAMPT levels in male rats [59]. The reason for this discrepancy is not clear. However, these studies mostly suggest that exercise training or electrical stimulation could be a tractable approach to increase NAMPT levels until pharmacological interventions are identified/improved.

Experiments elucidating the mechanisms regulating the exercise-mediated increase in NAMPT expression thus far implicate AMPK. Stimulation of AMPK is sufficient to increase NAMPT protein in skeletal muscle: 4 weeks of activating AMPK with the adenosine monophosphate (AMP) analog AICAR (5-aminoimidazole-4-carboxamide ribonucleotide) increased NAMPT expression. AICAR treatment in AMPK knockout mice did not increase NAMPT expression [57]. These experiments showed that AMPK activation is sufficient to increase NAMPT expression. However, a critical experiment showed that AMPK is not necessary for the exercise-induced increase in NAMPT expression in vivo: NAMPT expression was increased in endurance-trained AMPK knockout mice [57]. In the future, it will be interesting to determine the AMPK-independent mechanism of increased NAMPT expression in an effort to maximize NAD+ levels in muscle via exercise and/or pharmacological interventions.

### Other approaches to increase NAD+ and SIRT1 activity during aging

A likely benefit of increased NAD+ availability in muscle during aging is potentiation of SIRT1 activity. Many methods of enhancing SIRT1 activity have been discovered and shown to have positive effects on muscle healthspan. Activation of SIRT1 by a small molecule agonist, SRT2104, resulted in increased muscle mitochondrial size and better endurance in aged mice [60]. SRT2104 also reduced loss of muscle mass in the 2-week hindlimb suspension model of muscle atrophy [60]. Another method involves activating NADH-quinone oxidoreductase 1 (NQ01) with beta-lapachone [61]. NQ01 uses NADH as an electron donor and thus depletes NADH and increases the NAD+/NADH ratio [62, 63]. Aged mice fed with beta-lapachone showed increased oxygen consumption, increased heat generation, and improved skeletal muscle mitochondrial biogenesis [61]. Another approach to enhance NAD+ levels is to provide intermediates in NAD+ biosynthesis. Mice fed NMN for 12 months were more active and had improved oxidative metabolism without overt side effects [43]. The particularly interesting aspect of this study is that microarray analysis did not detect a common set of “NMN response genes” between different tissues. This suggests that the response to enhanced NAD+ metabolism is tissue specific. Similar to the above experiments, supplementation with NAM for 5 weeks enhanced SIRT1 activity in young and aged rats [64]. However, combining exercise training with NAM supplementation *decreased* SIRT1 levels [64]. NAM can inhibit SIRT1 and PARP-1 under stress conditions to preserve NAD+ levels. Thus, NAM supplementation may not be as effective as other methods of enhancing NAD+ levels and SIRT1 activity in aging muscle.

Modulating levels of the major NAD+ consumer PARP-1 have also been shown to be effective in maintaining muscle function during aging. In young mice, isometric exercise increases activity of both SIRT1 and PARP-1, but SIRT1 then binds PARP-1 and inhibits its activity [20]. Thus, young mice are protected from excess activation of PARP-1 and the potential for necrosis that NAD+ depletion can incur. In contrast, aged muscle has higher PARP-1 activity, lower NAD+ content, and lower SIRT1 activity, resulting in decreased performance. The higher PARP-1 activity is partially due to increased acetylation of PARP-1 by GCN5 [20]. NAD+ levels may also indirectly regulate PARP-1 activity. DBC1 (deleted in breast cancer 1) binds and inhibits PARP-1 in a NAD+-dependent manner in vitro [65]. Whether this occurs in skeletal muscle is unknown, but this could be an additional mechanism that regulates PARP-1 activity during aging. Inhibition of PARP-1 by injecting the inhibitor PJ34 in aged mice increased NAD+ content, SIRT1 activity, muscle mitochondrial biogenesis, and performance [20]. PGC-1alpha was not required, which raises the question of whether the increased mitochondrial biogenesis is due to stabilization of HIF-1alpha. Taken together, these results suggest that regulation of NAD+-dependent enzymes, such as decreasing PARP-1 activity, decreasing GCN5 activity, or activating SIRT1, could prevent or slow the age-associated decline in skeletal muscle mass and function.

### NR supplementation rejuvenates aged muscle stem cells (MuSCs)

It is easy to imagine that there could be crosstalk between the cellular pathways involved in lifespan extension. The mitochondrial unfolded protein response (UPRmt) is an adaptive pathway that mediates proteostasis in response to stress. Activation of UPRmt correlates with longevity in worms and mice [66]. Thus, it is perhaps not surprising that gene expression analysis of aged MuSCs showed reduced UPRmt, decreased cell cycle, and increased proinflammatory secretome gene expression [46]. NAD+ biosynthesis was found to feed into the UPRmt response. Nicotinamide riboside (NR) treatment increased levels of UPRmt proteins, increased MuSC number in young and aged mice, and quickened muscle regeneration after cardiotoxin-induced injury in both young and old mice [44]. Importantly, infiltration of fibro-adipogenic progenitors (FAPs) was decreased after injury in NR-treated mice. Knockout of SIRT1 in MuSCs abrogated the beneficial effects of NR, indicating that, in this context, NR acts through increased NAD+ and the ensuing increase in SIRT1 activity [46]. The ECM microenvironment provides a niche that regulates MuSC behavior [67]. As mentioned above, NAD+ plays a role in modulating organization of the ECM during muscle development [33]. Thus, one future direction would be to determine if NR supplementation affected MuSC responses by acting on the ECM in the MuSC niche. It would also be interesting to investigate whether NAD+ bioavailability feeds into other pathways that promote longevity.

## NAD+ in myopathies and dystrophies

**Fig. 6:**
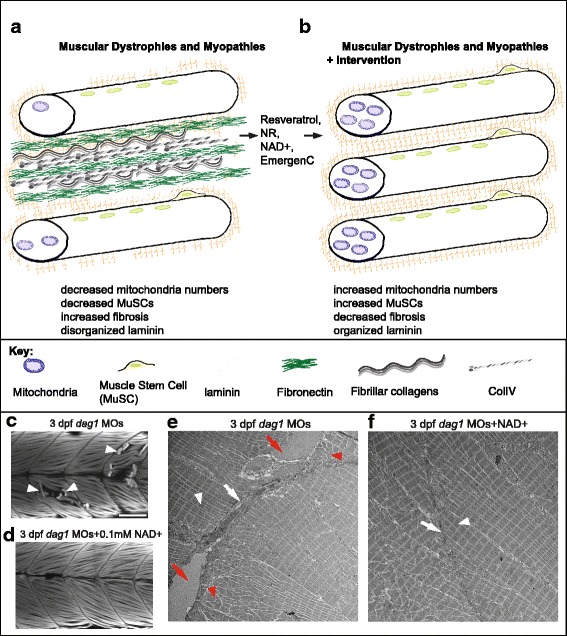
Cellular and molecular mechanisms of amelioration of muscle disease due to NAD+ repletion. (**A**, **B**) Model of pathological changes observed in animal models of diseased muscle (**A**) and the improvement in these disease phenotypes due to provision of dietary NAD+ precursors or NAD+ (**B**). (**C**, **D**) Anterior left, dorsal top, side-mounted, 3 dpf embryos stained with phalloidin to visualize actin. Fiber detachment is readily observed in *dag1* morphants (**C**, white arrowheads), whereas *dag1* morphants supplemented with NAD+ display less fiber detachment (**D**). (**E**, **F**) Transmission electron micrographs showing normal muscle ECM (white arrows), disrupted muscle ECM (red arrows), normal sarcomere structure (white arrowheads), and disrupted sarcomere structure (red arrowheads) in a zebrafish model of muscular dystrophy with and without NAD+ supplementation. (**E**) Dag1-deficient zebrafish. (**F**) Dag1-deficient zebrafish supplemented with NAD+

Normal aging and sarcopenia result in decreased muscle performance; however, some individuals lose muscle mass and function due to genetic muscle diseases. Muscular dystrophies and myopathies are stereotypically characterized by muscle wasting, weakness, and impaired locomotion (Fig. 6). Although significant progress has been made with regard to identifying disease causing genes and pathological mechanisms, these conditions are still incurable. One major challenge that remains is to understand if therapeutic crossover exists between the treatments for Duchenne/Becker muscular dystrophy (DMD) and the other types of muscular dystrophies. This is an important question because therapeutic strides are being made for the most common type of muscular dystrophy, DMD, but treatment for most other types of muscle diseases lags behind. Therefore, in the future it is critical to determine whether differences in cellular pathology affect treatment options. Given the critical role of NAD+ in muscle development, homeostasis, and aging, it is tempting to speculate that potentiating NAD+ biosynthesis could be a beneficial adjuvant therapy for a broad spectrum of muscular dystrophies and myopathies.

Resveratrol is a grape polyphenol that has received attention for its potential to extend lifespan. Resveratrol can directly activate SIRT1 [68] as well as indirectly activate SIRT3 through increasing the mitochondrial NAD+/NADH ratio [69]. Thus, resveratrol may have the potential to improve muscle function in aging and/or disease. Resveratrol was tested for possible efficacy in the *mdx* mouse model of DMD [70]. Thirty-two weeks of resveratrol supplementation resulted in reductions in both muscle mass lost and fibrosis. The decreased fibrosis presumably resulted from reduced transcription of collagen 1a1, 1a2, and fibronectin [70]. Resveratrol was provided in the diet; thus, it is not known in which tissues/cell types resveratrol functions to reduce fibrosis. Angiotensin-II is known to promote fibrosis via NAD(P)H oxidase-generated reactive oxygen species (ROS) in myoblasts [71]. These results suggest that myoblasts, in addition to fibroblasts and macrophages, can promote fibrosis. Given that CD45+ inflammatory cells were still observed in *mdx* muscle despite resveratrol treatment [70], it would be interesting to determine whether resveratrol reduced transcription of ECM genes in immune cells, myofibroblasts, or both.

Another method of increasing NAD+ biosynthesis was shown to be beneficial in the mouse model of DMD. NAD+ repletion with NR supplementation improved muscle function in *mdx* mice [44]. At least some of this improvement is likely due to the NR-mediated increase in number and function of MuSCs in *mdx* mice [46]. Taken together, these data suggest that NAD+ repletion, via multiple mechanisms, is a worthwhile avenue to explore in humans with DMD.

Could NAD+ repletion benefit other types of muscular dystrophies? As mentioned above, we showed that Nrk2b is necessary for normal muscle ECM development [33]. Laminin in the basement membrane was disorganized in Nrk2b-deficient zebrafish and rescued with exogenous NAD+. We found that muscle atrophy in a zebrafish model of primary dystroglycanopathy due to Dag1 deficiency is preceded by disorganized laminin in the muscle ECM. We asked whether exogenous NAD+ would improve laminin organization in Dag1-deficient zebrafish. We found that either supplementation with NAD+ or EmergenC, which contains vitamin B precursors to NAD+, improved laminin organization, reduced muscle degeneration, and improved swimming in Dag1-deficient zebrafish [72]. We identified the Integrin alpha6 and alpha7 receptors as well as intracellular localization of the focal adhesion protein Paxillin as downstream mechanisms. Using genetic mosaic analysis, we showed that the ECM microenvironment plays a critical role in maintenance of muscle cell-ECM adhesion and fiber viability and thus tissue homeostasis [72]. These results and others suggest that understanding the mechanisms downstream of NAD+-mediated ECM organization could lead to new therapeutic approaches (Fig. 6).

Increasing NAD+ bioavailability also appears to be beneficial for a myopathy. NR supplementation delays the progression of mitochondrial myopathy by stimulating the UPRmt as well as inducing mitochondrial biogenesis [73]. Given the dynamics of mitochondrial remodeling in muscle homeostasis and disease, it seems critical to understand mitochondrial dynamics in live organisms. Generation of tools that allow observation of mitoflashes in living zebrafish provided insight into how mitoflashes change in development and disease [74]. A mitoflash is a burst of ROS that mediates mitochondrial-cytosol/nuclear signaling [75]. It is not currently known whether or how mitoflashes affect the progression of muscle disease. Transgenic zebrafish expressing the biosensor mt-cpYFP [76] showed that mitoflashes are shorter early in muscle development but become longer as development proceeds [74]. Transgenic zebrafish modeling DMD showed the expected decrease in mitochondria numbers, although whether this reduction was due to decreased mitochondrial biogenesis was not investigated. Interestingly, the mitoflashes that were observed in zebrafish with muscle disease were longer than those of wild-type zebrafish [74]. The signaling events downstream of longer bursts of ROS are not known, but do provide an interesting avenue of investigation to pursue in terms of muscle disease pathology and progression.

The above data show that increasing NAD+ levels, whether by supplementing with resveratrol, NR, or EmergenC, is beneficial for multiple types of muscle disease. The mechanisms hypothesized to be responsible for ameliorating disease include increased: mitochondrial biogenesis, UPRmt, MuSC numbers, and laminin organization in the muscle ECM (Fig. 6). Increased NAD+ levels also correlated with reductions in both fibrosis and FAPs. However, none of these studies integrated or looked for connections between all of the different facets of muscle phenotypic improvement. The ECM microenvironment is involved in fibrosis as well as the health of muscle fibers and MuSCs. Thus, it would be interesting in the future to examine whether there is coordination between mitochondrial health and muscle ECM structure or whether these are two entirely separate mechanisms of NAD+ action. It also seems important to investigate whether NAD+ supplementation is beneficial across the spectrum of muscular dystrophies/myopathies to determine if there are muscle diseases that are resistant to NAD+ supplementation. There is one study suggesting that genotype may impact the response to exogenous NAD+. Dystrophic myoblasts (from *mdx* mice) showed an increase in ERK1/2 phosphorylation in response to exogenous NAD+, whereas control myoblasts did not [77]. The physiological significance of these results is not known, but they do highlight that NAD+ may have varying effects in different genetic environments. Thus, addressing whether activating NAD+ biosynthesis exhibits therapeutic crossover for multiple dystrophies and myopathies should be a priority. Understanding how mechanisms downstream of increased NAD+ cause certain muscle outputs should also be prioritized as this could provide a way to elicit desired muscle responses to NAD+ in different genetic backgrounds.

## NAD+ in muscle metabolic diseases

Skeletal muscle mass and function are not only critical for daily life but also whole-body protein metabolism and thus play a critical role in metabolic diseases such as obesity and type 2 diabetes (reviewed in [2]). Metabolic diseases are common and debilitating conditions. Given the roles of skeletal muscle and NAD+ in metabolic regulation, it is possible that NAD+ supplementation could improve metabolic health.

PARP-2 is a major NAD+ consumer and thus indirectly affects SIRT1 activity and metabolic health by modulating NAD+ levels [21]. High fat diet-fed mice have elevated PARP-2 expression in skeletal muscle, which would be predicted to act as a NAD+ sink and lower SIRT1 activity [21]. Therefore, PARP-2 inhibition is a potential strategy to augment NAD+ levels in skeletal muscle in the context of obesity. Mohamed et al. conducted a microRNA (miR) screen to identify potential miRNAs that modulate PARP-2 expression. They identified miR-149 as necessary and sufficient to decrease PARP-2 expression, increase SIRT1 activity, and increase mitochondrial biogenesis in C2C12 cells [21]. Taken together, these results suggest that targeting either PARP-2 expression or enhancing miR-149 expression could be potential approaches to promoting metabolic health via NAD+ repletion in skeletal muscle.

Interestingly, boosting NAD+ levels by 50% in mouse skeletal muscle via tissue-specific overexpression of NAMPT did not induce mitochondrial biogenesis or protect these mice from the negative metabolic consequences of a high fat diet [78]. Therefore, augmenting NAD+ in tissues other than striated muscle is necessary for promoting mitochondrial function and oxidative metabolism [78]. The tissues in which NAD+ repletion is required for whole body metabolic improvement are unknown and are important targets to elucidate for metabolic therapies.

Human trials that analyze the consequences of increasing NAD+ on muscle are limited. However, one recent multicenter trial provided patients with type 2 diabetes either placebo or the NAD+ precursor acipimox for 2 weeks [79]. Muscle biopsies allowed for examination of muscle respiration and gene expression after the treatment. Although mitochondrial DNA content did not differ between the treatments, respiration was higher in acipimox-treated muscle. Upregulation of gene sets that have been previously positively correlated with NAD+ supplementation were observed in this case [79]. While there were side effects to this particular type of NAD+ augmentation in these type 2 diabetics, these data suggest that roles for NAD+ in skeletal muscle and metabolic health are conserved in humans. These data also support the promise of pharmacologically or enzymatically modulating NAD+ biosynthesis and/or utilization as a therapy for many human genetic and metabolic disorders.

## Future perspectives

Why is it important to elucidate downstream mechanisms of NAD+ action if, in general, the studies presented here all show benefits to NAD+ supplementation? There is correlative evidence that increased NAD+ can be deleterious in the context of multiple muscle cancers. Higher NAMPT expression levels correlate with increased tumor grade for leiomyosarcomas, as well as embryonal, alveolar, and pleomorphic rhabdomyosarcomas [80]. Thus, it is critically important to learn downstream signaling pathways affected by NAD+ signaling such that the benefits of NAD+ can be leveraged without potentially deleterious side effects. This is especially true because NAMPT expression not only correlates with tumor grade for muscle cancers but also correlates with invasion, metastasis, and chemotherapy resistance for gastric, thyroid, and prostate cancers [81–83]. Although current studies are focused on developing NAMPT inhibitors that are administered along with nicotinic acid to help alleviate toxic side effects of NAMPT inhibitors, it is likely that identifying downstream targets in normal and cancerous cells would lead to improved efficacy with fewer side effects.

## Conclusions

It is clear that NAD+ plays a beneficial role in muscle health. The mechanisms underlying promotion of muscle development and homeostasis by NAD+ are best understood in the context of sirtuin regulation in the nucleus, mitochondria, and cytosol. The role of NAD+ in other cellular compartments—particularly the vesicular and membrane proximal compartments—in muscle health is currently understudied. Future research will likely delve into not only the mechanisms of NAD+ action in these different compartments but also the interplay and signaling between NAD+ pools within and between cells.

## Abbreviations

ADP: Adenosine diphosphate
ADPR: Adenosine diphosphate-ribose
AICAR: 5-aminoimidazole-4-carboxamide ribonucleotide
AMP: Adenosine monophosphate
AMPK: Adenosine monophosphate kinase
ART: ADP-ribosyltransferase
ATP: Adenosine triphosphate
*C. elegans*: *Caenorhabditis elegans*
C2C12: Immortalized mouse myoblast cell line
cADPR: Cyclic ADP-ribose
CD45: Cluster of differentiation 45
Dag1: Dystroglycan
DMD: Duchenne/Becker muscular dystrophy
DNA: Deoxyribonucleic acid
ECM: Extracellular matrix
EDL: Extensor digitorum longus
ERK1/2: Extracellular signal-regulated protein kinase 1/2
FAP: Fibro-adipogenic progenitors
GCN5: Histone acetyltransferase GCN5
H2O2: Hydrogen peroxide
HIF-1alpha: Hypoxia inducible factor-1alpha
IL-6: Interleukin-6
ITGA7: Integrin alpha7
ITGB1BP3: Integrin beta1 binding protein3
L6: Immortalized rat skeletal myoblast cell line
*lamb1*: Laminin beta1
*lamc1*: Laminin gamma1
*mdx*: Mouse DMD model
MIBP2: Muscle integrin binding protein2
miR: microRNA
mNKO: Muscle NAMPT knock out
MuSC: Muscle stem cell
NA: Nicotinic acid
NAAD: Nicotinate adenine dinucleotide
NAADP: Nicotinic acid adenine dinucleotide phosphate
NAD+: Oxidized nicotinamide adenine dinucleotide
NADH: Reduced nicotinamide adenine dinucleotide
NADPH: Reduced nicotinamide adenine dinucleotide phosphate
NADS: NAD+ synthase
NAM: Nicotinamide
NAMN: Nicotinate mononucleotide
NAMPT: Nicotinamide phosphoribosyltransferase
NAPRT: Nicotinate phosphoribosyltransferase
NAR: Nicotinic acid riboside
NEFA: Nonesterified fatty acid
NMN: Nicotinamide mononucleotide
NMNAT: Nicotinamide nucleotide adenylyltransferase
NQO1: NADH-quinone oxidoreductase 1
NR: Nicotinamide riboside
NRK1/2: Nicotinamide riboside kinase 1 and 2
Nrk2b: Nicotinamide riboside kinase 2b
OAADPR: O-acetyl-ADPR
OXPHOS: Oxidative phosphorylation
PARP: Poly (ADP-ribose) polymerase
PCAF: p300/CBP-associated factor
PGC1-alpha: Peroxisome proliferator-activated receptor gamma coactivator 1-alpha
PJ34: PARP inhibitor VIII
QA: Quinolinic acid
QAPRT: Quinolinate phosphoribosyltransferase
Redox: Oxidation-reduction
ROS: Reactive oxygen species
SIRT: Sirtuin
SR: Sarcoplasmic reticulum
SRT2104: Selective activator of SIRT1
TFAM: Mitochondrial transcription factor A
UPRmt: Mitochondrial uncoupled protein response
VHL: Von Hippel-Lindau tumor suppressor
